# Should condoms be available in secondary schools? Discourse and policy dilemma for safeguarding adolescent reproductive and sexual health in Rwanda

**DOI:** 10.11604/pamj.2018.31.173.16549

**Published:** 2018-11-13

**Authors:** Germaine Tuyisenge, Celestin Hategeka, Ruben Alba Aguilera

**Affiliations:** 1 Department of Geography, Simon Fraser University, Burnaby, BC, Canada; 2 Centre for Health Services and Policy Research, School of Population and Public Health, Faculty of Medicine, University of British Columbia, Vancouver, BC, Canada; 3 Collaboration for Outcomes Research and Evaluation, Faculty of Pharmaceutical Sciences, University of British Columbia, Vancouver, BC, Canada; 4 Civil servant at the European Commission working at the Delegation to the African Union, Addis Ababa, Ethiopia

**Keywords:** Adolescent reproductive and sexual health policy, sex education, secondary school students, claims-making, teen pregnancy, Rwanda

## Abstract

**Introduction:**

As a response to challenges associated with adolescent reproductive and sexual health, policy makers in Rwanda have instituted preventive measures against risky sexual behaviours among adolescents. There is an ongoing debate on whether condoms should be made available in secondary schools to minimise risks related to unprotected sex in the context of a growing number of unintended pregnancies among school girls. This paper aims to examine the proposal of condom provision in Rwandan secondary schools through the analysis of policy narratives and the claims-making process.

**Methods:**

A narrative policy analysis was used to understand the claims and counter claims surrounding the debate on the provision of condoms in secondary schools. Documents that were consulted include: the national reproductive health policy, the girls' education policy, the national behaviour change and communication policy for the health sector, the Rwanda national policy on condoms, the adolescent sexual reproductive health and rights policy and the Rwanda family planning policy.

**Results:**

Social and cultural norms in the Rwandan context consider adolescent sexual practices as immoral and thus reject the idea of providing condoms in secondary schools. However, some stakeholders promoting reproductive health suggest that ignoring that some adolescents are sexually active will prevent them from accessing appropriate reproductive and sexual health protective programmes. Consequently, adolescents will be exposed to risky sexual behaviours, a situation which may be counter productive to the overarching goal of safeguarding adolescent sexual health which might impact their long-term education goals.

**Conclusion:**

Making condoms available in secondary schools evokes different meanings among the debaters, underscoring the complex nature of the condom provision debate in Rwanda. This paper calls for a revision of policies related to adolescent reproductive and sexual health in order to answer to the issues of risky sexual behaviours among secondary school students.

## Introduction

Adolescent sexual and reproductive health is an issue of immense concern globally and is part of the overall objective to achieve universal access to efficient healthcare services. Social, cultural, religious, and economic factors have an important impact on the progress of sexual and reproductive health among adolescents [1, 2]. Unsafe sexual activity-i.e, sexual activity that exposes either of the partners to negative outcomes such as diseases or unintended pregnancy, human immunodeficiency virus and acquired immune deficiency syndrome (HIV/AIDS) and other sexually transmitted infections (STIs) are major risks for high mortality and morbidity. The rates of such infections are especially high in low-and middle-income countries (LMICs), hence calling for combined effort to reduce them [2]. The World Health Organization (WHO) reports that two million youth between 10 and 19 years are infected with HIV around the world [3]. Moreover, the adolescent birth rate for adolescent girls aged 15-19 was 44 per 1000 in 2015 [4]. Teenage pregnancy presents risks to both the baby and mother, with increased pregnancy complications to the mother as well as high risk of disease and death to both the baby and mother. Teenage pregnancy may lead to unsafe abortion, especially in countries where abortion is illegal and or practiced in no-safe conditions due to poor hygiene and inadequate equipment [5]. Teenage pregnancy has also been cited as a major cause of school dropout among young girls [6].

In Rwanda, the most recent Demographic and Health Survey (DHS) (2015) indicated that 7% of teenage girls between 15 and 19 years have either already experienced child bearing or were pregnant [7]. The same document showed that the proportion of childbearing teenagers, which was 11% in 1992 showed a decrease of 4% in 2000 and a decrease of 3% in the year 2005. However, this rate increased again in the year 2010 and was at 6%. No studies have been done to explain the decrease and increase in teen pregnancy rates over time. The Rwanda Ministry of Gender and Family Promotion (MIGEPROF) reported an unprecedented high number of 17,750 of reported pregnancy in the year 2016 for teen girls between 16 and 17 years, translating into at least 47 deliveries by teen mothers per day [8]. These data highlight the involvement of adolescents in non-protected sexual activity and a lack of use of other contraceptive methods, calling for action by stakeholders in sexual reproductive health in Rwanda. The meanings that hinge on morality, birth control, social protection of present and future generations, child rights, HIV prevention, sex education, sexual health, gender, reproductive health, and social responsibility, to name a few have been ascribed to a controversial proposal on the provision of condoms in high schools.

### The proposal of condom provision in secondary schools in Rwanda

Debates on the proposal of providing condom in secondary schools raised great attention in the late 2000s, promoted by the Health Development Initiative (HDI), a local non-government organization (NGO), and supported by a number of other NGOs in the health sector. The proposal was in response to the need to protect the students in secondary schools against STIs and unintended pregnancies. HDI has been operating in Rwanda since 2005 to promote family and community health, policy monitoring and advocacy by targeting women, youth, children and other vulnerable groups through community health education. Due to socio-cultural and religious influences, discussions on sexual and reproductive health are still taboo in many Rwandan families. In schools, HDI conducts discussions with students on Sexual Health and Reproductive Education (SHARE), with the aim to ensuring that secondary school students are educated and able to protect their sexual and reproductive health. Several initiatives have been put in place by the Government of Rwanda (GoR) and other relevant stakeholders in adolescent reproductive and sexual health to promote safe and culturally acceptable sexual behaviour among youth. Institutions such as the Ministry of Health (MoH), Ministry of Education (MINEDUC), Ministry of Youth (MINIYOUTH) and MIGEPROF along with international and local NGOs collaborate with a wide range of civil society organizations to implement such initiatives and ensure their progress. For example, community health workers (a program by the MoH) conduct regular outreach interventions by providing community education on sexual and reproductive health to adolescents. In schools, MINEDUC has emphasized reproductive health education programs in the education curricula and encouraged parents to discuss reproductive health with their children while keeping in mind social and cultural values [9]. Likewise, MINIYOUTH has put the emphasis on the provision of youth-friendly reproductive and sexual health programs such as *Dushishoze Centres* for youth friendly sexual and reproductive health dialogue and services in rural areas.

Despite the introductions of such initiatives, the alarming number of pregnancies among school girls across the country indicates that students are sexually active [8]. A behaviour surveillance conducted in 2009 on youth (aged 15-24) indicated that these youth were sexually active [10] and a survey conducted in three rural schools of Rwanda indicated that 44% of students reported to be sexually active and among them, only 36% used condoms regularly [11]. The Rwanda National Policy on Condoms (RNPC), established in 2005, stated that accessible condoms would help to prevent sexually transmitted diseases (STDs) (particularly HIV/AIDS) and unwanted pregnancies [12]. Yet, 13 years after the adoption of the policy, students are not provided with condoms in secondary schools. Rwanda's DHS (2010) states that only 13.3% of women and 10.7% of men aged 15 to 19 used condoms during their last sexual intercourse [10]. In order to explore the discourse surrounding the proposal of condom provision in high schools, this paper aimed to address the following questions: Who are the participants in the adolescent sexual and reproductive health discussions in Rwanda in the context of condom provision in high schools? What are the dominant policy claims of various individuals groups in the condom provision debate? How do these policy claims shape the dynamics of the condom provision debate? In particular, what are the stories, non-stories and meta-narratives that emerge from the debate? This paper argues that both proponents and opponents in the debate are struggling to make their world views and narratives the dominant issue in the debate.

## Methods

A Narrative Policy Analysis (NPA) was used to understand the claims and counter claims surrounding the debate on the provision of condoms among secondary school students. This approach was used due to its nature that helps to understand complex policy debates where there is uncertainty, complexity and confusion surrounding an issue [13, 14]. Using tools from literary criticism, the method helps to understand the relationship between different narratives surrounding the issues and to seek a meta-narrative which suggests a reframing of the situation from which progress can be made by more conventional analysis. As a method for analysing policy it is suitable in situations where conventional policy analysis fails [14]. The stories used provide an important basis for research, or narrative analysis. NPA is based on the view that people, faced with the necessity of “constructing and representing the rich and messy domain of human interaction”, tend to “organize [their] experience and [their] memory of human happenings mainly in the form of narrative-stories, excuses, myths, reasons for doing and not doing, and so on” [15].

A review of Rwandan policy documents on adolescent reproductive and sexual health was conducted. These included the policies on reproductive health, girls' education, national behavior change communication, family planning, adolescent sexual reproductive health and rights 2011-2015 as well as the National Policy on Condoms [12, 16-20]. The authors consulted with the actors in adolescent reproductive health in Rwanda to make sure that all relevant policies are examined for the purpose of this study. The analysis of policy document was complemented with narrative analysis of public policy discussions on condom provision in Rwanda. For ease of access of claims-making by the actors in the debate, this paper was restricted to stories in the electronic media. Different narratives were selected from electronic media (private and public) that reported on the proposal of condom provision in secondary school. Hannigan (2006) notes the importance of media in raising up problems to be part of political process [21]. Media helps the public to get awareness of information about complex issues that need attention from people in different categories.

The searched materials included the websites of NGOs in Rwanda, the websites of the ministries of health, education gender promotion and youth along with Rwandan electronic media from September 1, 2011 through January 31, 2018, corresponding to the time where HDI had initiated the debate about distributing condoms in secondary schools until the time of writing the article. Our search used terms including “condom provision and secondary/high schools and Rwanda”, “condom provision and secondary/high schools and Rwanda", “condom and high/secondary school and Rwanda”, “teenage pregnancy and high/secondary school and Rwanda”, “HIV/AIDS and high/secondary school and Rwanda”, “school dropout and pregnancy and Rwanda”, “adolescent health and high school and Rwanda”. All authors, fluent in English, independently evaluated the electronic materials from websites in English. Materials in Kinyarwanda language were evaluated by two of the authors who are native speakers of the language.

Studies that fulfilled the following a priori eligibility criteria were included: if the electronic material (1) was from an original source; (2) reported an issue specifically related to the condom provision policy discussion (3) reported on any one or more of the following issues: teenage pregnancy, adolescent health, reproductive health, pre-marital sex, sex education, (4) presented information on HIV/AIDS risk to teenagers. Thirty electronic materials were retrieved including 21 private and nine public articles. All the articles were reviewed in-depth and only ten articles that contained adequate information for the purpose of this paper were used.

After selection of the electronic materials on condom provision, the narrative policy analysis stepwise procedure proposed by Roe (1994) was used [22]. Initially, dominant stories (those stories that are repeatedly told) by proponents and opponents in the debates were identified. Next, non-stories (stories that do not have a beginning, middle or an end) and counter-stories (stories that run counter to the dominant policy narratives) were identified. Stories were then contrasted and combined to form a new story in the form of a metanarrative (alternative to consensus) in the thirds step. Finally, the metanarrative and how it recasts the condom provision policy problem was examined. The ethics approval was not required because only data available in the public domain were analyzed.

## Results

**Characteristics of the search materials:** Apart from the five policy documents, about 30 electronic materials were retrieved and all of them were reviewed in-depth. Twelve materials/articles/news reports fulfilled our a priori inclusion criteria for this narrative policy analysis. Ten of these studies provided adequate information based on our search terms and inclusion criteria. Twenty materials were excluded for various reasons (e.g. duplication, irrelevant information on condom distribution). Table 1 summarizes the nature of selected electronic media.

**Table 1:** Electronic media retained for review and language of publication

Date published	Source	Language
November 4, 2011	Kigalitoday.com	Kinyarwanda
November 30, 2011	Voanews.com	English
January 30, 2012	Thenewstimes.co.rw	English
January 25, 2013	Igihe.com	Kinyarwanda
June 12, 2013	Allafrica.com	English
February 13, 2014	Igihe.com	English
February 7, 2015	Theeastafrican.co.ke	English
August 14, 2016	Makuruki.rw	Kinyarwanda
December 23, 2017	Kigalitoday.com	Kinyarwanda
January 29, 2018	Umubavu.com	Kinyarwanda

**Description of relevant policies:** This section gives a brief description of policies that have been selected as relevant to the dialogue of condom distribution among secondary schools. These policies were implemented by the GoR through different institutions as a result of the country being a member of other international treaties that aim to protect and promote safe reproductive health.

***National reproductive health policy:*** Established in 2003, the National Reproductive Health Policy focuses on six components: safe motherhood/child health, family planning, prevention and treatment of genital infections (STIs and HIV/AIDS), adolescent reproductive health, prevention and management of sexual violence, and social change to increase women's decision-making power [16]. In regard to youths, the reproductive health policy emphasizes on the role of information, education, communication and access to HIV/AIDS testing services. In schools, adolescent reproductive health education is included in teaching curricula, from primary schools to higher education and different organizations participate in reproductive health education (church based and anti-AIDS clubs among others). These are established in schools, and at the community level, with the aim of increasing awareness of reproductive health and prevention against unsafe sex consequences. The policy does not put in light the means of access to reproductive health for youth, even though the policy document reports that 25% of teens below 18 years are sexually active.

***Girls' education policy:*** In Rwanda, the rates of school dropout are higher among females. The main causes of school dropout in girls, are household demands, unintended pregnancy and prioritizing boys' education over girls' education [18]. The girls' education policy was implemented in 2008 with an overall objective to guide and promote sustainable action aimed at the progressive elimination of gender disparities in education. The strategies to achieving the girls' education policy include access, quality/achievement and retention/completion. Preventing teen pregnancy is one of the means to achieve girls' access and retention/completion in schools.

***National behaviour change communication policy for the health sector:*** Implemented in 2006, the objective of this policy is to help in the improvement of public health, by preventing the transmission of communicable diseases. In schools, MINEDUC and MOH collaborate to provide health promotion through behaviour change communication (BCC) [17]. The message they give includes information about reproductive health and awareness on how to prevent against unsafe sex, emphasizing on abstinence, which is the sure mean of protecting against unintended pregnancy and HIV/AIDS.

***Rwanda national policy on condoms:*** Different government institutions, NGOs and community sectors participated in the establishment and implementation of the policy in 2005 [12]. *"The vision and goal of the condom policy is that sexually active individuals and couples recognize their risk of HIV/STI transmission as well as unwanted pregnancies and take measures to avoid risk exposure for themselves and their partners' (p.7).*Information and education are the key starting points in prevention against HIV/AIDS and unintended pregnancies. Its goal is to improve common understanding of condom use in the promotion of sexual and reproductive health. Condom use plays an important role in insuring safe sex. High risk groups that are targeted by this policy are: sex workers, professional drivers, refugees, migrant laborers, workers in tea and coffee plantations, persons in uniform (police and armed forces), prisoners, students, women without partner, and people living with HIV/AIDS.

***Family planning policy:*** In order to respond to the demographic dividend for a small country like Rwanda with the highest population density in Africa (436 persons/ square km), a policy to contribute to birth limiting and spacing seem rather imperative. As a result, the family planning policy was adopted in 2012 to answer to population growth and as an indirect channel to achieve other development goals (first the Millennium Development Goals followed by the Sustainable Development Goals) which targeted poverty reduction and development, gender promotion, promotion of education especially for girls, reduction of maternal and infant mortality, among others [20]. Additionally, the policy was implemented to insure physical access to contraception by increasing its availability among communities. By this policy, community health workers were able to sensitize the population and provide them with selected contraceptives (pills, injections, condoms) in their villages without having to travel distances to health facilities. Through the family planning policy, top priority for condom distribution in public and private sectors was given to make sure they would be easily available to all, with no discrimination whatsoever. The policy targeted the reproductive age group (15-49) for both male and female, with an emphasis on youths. This would help to prevent teenage pregnancy, stressing the importance of dual protection for both pregnancy and STIs.

***The adolescent sexual and reproductive health and rights policy:*** Along with other countries who participated at the Cairo (Egypt) international conference on population and development in 1994, Rwanda acknowledged adolescent reproductive health as a right. In order to make this a priority on its agenda, the MoH implemented a policy that would promote reproductive health for teens and young adults (ages 10-24) [19]. Despite the complex and sensitive nature of sexual reproductive health for this specific age group in the Rwandan society as a result of traditional norms, the policy aimed to take into consideration the principles of sexual and reproductive health: *1) good customer care, 2) greater integration of related services within existing health services to suit adolescents' needs, and 3) confidentiality as the three main underlying principles to enhance these services' acceptability and accelerated uptake by adolescents and young adults (p.14).* To this end, the MoH calls for adolescent and youth partnership for the success of this policy.

**Policy claims, arguments and counter claims on the distribution of condoms:** This section is divided into two sections representing the claims and arguments of proponent and opponent groups on condom distribution among secondary schools. In order to support these arguments, different quotes have been selected from online magazines, news articles, and government and non-government organization sites. Quotes were from community members and stakeholders in youth reproductive health: representative of the civil society, students, parents, teachers, religion representatives, government and non-government officials. Selected materials represented views from individuals of different age categories ranging from youth to elders, with both male and female gender groups represented. In addition, the materials used in this study represent views from individuals from both rural and urban settings ranging across different spectrum of education. Table 2summarizes the main claims from opponent and proponent groups and the role of stakeholders in youth reproductive health.

**Table 2:** Role of participants in the condom distribution debate as reported by the electronic media

Stakeholders	Role in society	Proponents	Opponents
Government officials	Policy makers	face the reality that students are sexually active and protect them	students are underage to be sexually active
NGO individuals	Policy makers/advocates	students have to benefit from all the reproductive health programs It is their right	
Faith based organizations	Civil society		against extramarital sexual behaviors
Students	Civil society	we have a right to be protected from unwanted pregnancy and sexually transmitted diseases	students have to abstain until they finish school
Parents	Civil society	our role is to protect students	our role is to direct students in the right direction
Teachers	Civil society	protection is most important	temptation to students who are not sexually active
Elders	Civil society	face the reality and enhance protection measures	sexual behaviours among youth is immoral, they have to keep values of abstinence

**Claims from proponent groups:** Following the growing rates of teenage pregnancy in the country, the promoters of condom distribution among secondary schools argue that it is now time to accept the fact that some adolescents are sexually active. This will help to protect them against any negative outcomes of unprotected sex and eventually reduce school dropouts that result from teen pregnancy. A number of teachers, students, parents, civil society and non-government organizations support this initiative as a measure to protect the youth. For example, a parent, based on the growing figures of teen pregnancy said: *“If reports from the Ministry of Education indicate that the dropout rate of female students is attributed in part to pregnancies, as a parent what comes to your mind?. let's face the reality, put in place protection measures to avoid the worst, (Parent, Male (M), Gasabo)”.* In support of this, a teacher argued that taboos must be lifted and recommended that people speak about condoms to school children openly: *“I think we should stop burying our heads in the sand. It's not a secret that some of our children here in the city start having sex at a very young age: 11, sometimes even 10. Is it because their parents and teachers do not tell them about the dangers they're exposing themselves to? Catholic preachers, priests and imams have all been preaching about it for years, but nothing has changed. And every passing day brings even more temptations that make our children fall into such traps (Secondary School Teacher, M, Kicukiro).”*

To buttress the stance above, another teacher shared a similar opinion: “We should educate these young people about condom use and avail them because either way, they engage in sexual intercourse, so the earlier we teach them the better.I don't think this will necessarily push them into early sex because emphasis will be put on the essence of sexuality so that the students understand the rightful purpose of sex and condoms. (Secondary School Teacher, Female (F), Nyarugenge)”. HDI points out that condom access has to be part of the behaviour change package provided to students, and that condom distribution would be an answer to students' complaints on risky sexual behaviours. In this context, an NGO official illustrated that: “It is important for young people to be equipped with knowledge of and access to condoms. Some of our secondary school children have sexual relations with their classmates or teachers. If we want to build a healthy generation of Rwandans, capable of working towards the country's development, condoms should be made available in schools (NGO official, F, Nyarugenge)." Some students also considered condom distribution as a solution to girls school dropouts. ”I think condom distribution is OK since I see girls getting pregnant and dropping out of school.As long as they teach students how to use them properly then we shall not be faced with such problems again. (Secondary School Student, F, Gasabo)." Another student thinks that whether made available to them or not, condoms among students are needed. “Students, especially in mixed [boys and girls] schools, are sexually active and even if condoms are not available inside boarding schools, students should be permitted to go out and buy them (Student, M, Kicukiro).”

However, most of individuals recognise the fact that condoms should come as a last option in the preventive measures. *"Condoms must be recommended as a last resort, in cases when lust trumps logic. (Secondary School Teacher, F, Kicukiro).”* Some parents share the same view: *“I'm sure every society would wish to preach abstinence first. But the fact is that abstinence works best for people who are religious, or people who uphold their morals and have a higher degree of self-restraint. People are not all the same. We don't fight the same way and everybody needs different solutions to their problems. For that reason, condoms were invented to save people who would rather not abstain. Students also have a right to choose the best option to protect them from the AIDS pandemic (Parent, M, Gasabo, Kigali)”.* The claims above highlight the recognition of distribution of condoms as a last resort solution to protecting and promoting the sexual and reproductive health rights of the youth in secondary schools. However, there were counter claims that sought to erode the legitimacy and intrinsic value of the arguments of proponents.

**Claims from opponents groups:** In a conservative country like Rwanda, religious institutions such as the Catholic Church, occupy an important role in society and are responsible for the management of the highest number of secondary schools. As a result, they do express their position regarding extramarital sexual relationships in very strong terms. Moreover, institutions and society members who do not support the idea of condoms in secondary schools argue that students are underage to be sexually active. This group claims that secondary school students would rather benefit from reproductive health education, as a response to fight against HIV/AIDS and unwanted pregnancies. In this context, a member of the government said: *“As the government, our position is to encourage reproductive health education in our secondary schools in order to raise awareness among our youth on the dangers of underage sex, not to distribute condoms. (Government official, F, Kicukiro)”* Those who support the same idea emphasize school sensitization campaigns that do not mention the word “condom" in front of the students. *“It will (the use of condoms) amount to giving our children the go-ahead. It will incite sexual depravation even in those children not yet thinking about sex, (AMuslim, M, Nyarugenge)”* In faith-based schools, measures against sexual related behaviours are very strict and students are given serious sanctions when involved in such behaviours, including being expelled from school. This is exemplified in the following quotation: *"Students involved in sexual intercourse are fired from the school. We educate children not couples. (School Principal, F, Nyarugenge).”* Some students also object to the idea of condom distribution among schools: *“By simply availing condoms in schools it encourages people who would normally abstain to feel open about having sex (Student, F, Nyarugenge)”.* This shows a desire to protect those who are not sexually active rather than protecting all the students, as emphasized by this school leader: *“When students get pregnant, it means they have failed to behave. Therefore, let us not only look at the few to bring in issues that will also lead others into the same trap (Teacher, M, Kicukiro).”*

Individuals from this coalition stand on the fact that education is the key to the problem, and different groups have to be involved to promote adolescent reproductive health through education (students, parents and educators). *“I don't believe in condoms being distributed in secondary schools. It's a no go zone. The children are, in the first place, not mature enough to know how to use condoms.We should promote abstinence instead, and introduce condoms at a higher level - say universities and other higher institutions of learning (Teacher, M, Gasabo).”* Others claim to keep social norms and cultural value, by showing the youth in the right direction: *“We are tutors with the task of humanizing children who will serve the future generations and the country.so giving them condoms would be undermining their moral values that we want them to acquire (Teacher, F, Kicukiro).”*This is supported by some parents, who have to make their children's social values a success *“To say that condoms be introduced to these young children means we have lost our sense of direction and morals. We should emphasize postponement of sexual activity by encouraging these young people to embrace abstinence. How do I start encouraging my young girls to engage in sexual activity instead of concentrating on their academics (Parent, F, Gasabo)”* This is strongly supported by an elderly person, when asked about the issue *“I would even remove my children from any school that made condoms available to students.Students should instead be provided with guidance to abstain rather than being told to use condoms, which is reserved to only married couples (elder, M, Gasabo)”* For the opponent group, sexual involvement by adolescent is not only considered as immoral by religious preaching but also it is against the conservative traditional norms of the Rwandan society. Youth are restricted from being engaged in any sexual relationship. This group considers it to be a failure of the religions or elders if youth are to be provided with condoms. They insist on educating the youth on abstinence as the only way to bring back the society to its traditional norms regarding sexual behaviour.

## Discussion

There is still a lack of research on the use of condoms and their provisions in secondary schools, especially in Sub-Saharan Africa where most traditional norms prohibit adolescents to be sexually active. In Rwanda, the cultural beliefs disapprove any outside marriage sexual behaviour and encourage chastity among adolescents. Youth are considered to be underage to be sexually active and providing them with condoms is considered as a lack of societal norms and would mean that parents have failed their education responsibilities towards their children. Due to socio-cultural and religious influences, sexual and reproductive health discussions are still taboo in most Rwandan families. Parents prefer to leave the task of sexual and reproductive health education to teachers [23]. Fonner *et al.*, (2014), acknowledges the role of sex education among youth as an important key to prevent risky sexual behavior [24]. They encourage abstinence as the privileged way to prevent against HIV/AIDS and unwanted pregnancies. The authors also emphasize on the role of social norms in influencing sexual behavior, and suggest that there should be an attempt to modify them in favor of non-risky sexual behavior. This is stressed by Lo, Lowe and Bendavid (2016), who suggest that abstinence has not been effective as an only method to promoting reproductive health among youth in sub-Saharan Africa [25].

This is supported by individuals who agree with the provision of condoms in secondary school. They want to face the reality and danger that the youth encounters if not availed with protective methods. Their argument is that adolescent sexual behavior are risky as long as condoms are not introduced in school, as addition to the protective package that is already provided to students [21]. Studies conducted in different schools of the United States where condoms are distributed, did not show an impact on the increase of sexual activity among youth. They, however, showed an increase in the use of condoms among teens [26, 27]. A comparison study done in Massachusetts secondary schools where condoms where available and where they were not, concludes that the availability of condoms in schools does not initiate sexual activity among students who are not sexually active [28].

Even with such evidence, it is still difficult to support the proposal of condom provision in many developing countries, where cultures encourage chastity and promote moral values in regard to sex before marriage. South Africa is an example to this regard. Despite the country's high rates of HIV/AIDS worldwide with 35 % of new infections for youth aged 15-24, the policy of making available condoms in secondary schools is still unclear. The decision of condom provision in schools is left up to the schools and the members of the community where the school is located [29]. With this, the conservative culture in many communities of South Africa can be a barrier to encourage the condom provision despite the existence of a government policy.

It is important to acknowledge that different socio-economic factors play a big role in youth initiation to sexual activity and these may be different in developed and developing countries [30]. Thus, it is difficult to apply the conclusion from the above-mentioned studies where condoms are distributed in secondary schools to students in developing countries. However, scientific evidence supports that the effective use of latex condoms, the most common, reduces the risk of STDs and HIV/AIDS and the risk of pregnancy at 98% [31]. While most countries are making progress in regard to the pandemic of HIV/AIDS with a decreasing number in new infections and deaths due to HIV, countries like Nigeria, Zambia, the Philippines and Indonesia, where condoms are considered as illegal or not supported by official leaders, have shown a slow decrease if not an increase in the prevalence of HIV/AIDS [32]. Manuel (2005) argues that the access to condoms has been reported to meet considerable challenges among youth, such as social attitudes regarding sexual health, economic access and gendered factors [33].

When applied to secondary school students, this seems to be a very complex issue to deal with in the context of Rwanda, especially due to the fact that, even, among same category of people (age, gender, education, rural/urban setting), the problem is not seen from the same perspective. Uncertainty is found in different stakeholders of sexual and reproductive health groups: there are pro and opponents among youth, parents, teachers, elders, and government officials. Only religious groups stand for the same claim [34]. Religion plays an important role in shaping social and cultural norms in Rwanda, and religions, especially the Catholic Church, have an influential role in the education system in Rwanda.This shows the interrelation of science and politics, which takes antagonistic directions, when it comes to deal with cultural and social issues [35-37]. However, there is a need of a compromise to promote adolescent reproductive health. Concerned institutions are urged to frame a policy that would be seen as a solution for the issues raised by the two coalitions [22]. More data is needed on adolescent sexual reproductive health from schools and stakeholders institutions in order to better explore the issues surrounding risky sexual behaviours among adolescents, analyse the existing initiatives that have been put in place and explore how they could be strengthened in order to promote healthy sexual and reproductive behaviours among secondary school students.

**Study limitations:** This study analysed online materials, an approach that may see interpretations distorted by subjectivity as to an understanding of the key issues, subject manipulation, self-selection of target audience, and generalisation of results. Additional research including a representative sample of respondents and more online materials would be needed to reduce these limitations.

## Conclusion

Condom provision in secondary schools evokes different meanings among the debaters. This underscores the complex and messy nature of the condom provision debate in Rwanda. Rwanda's socio-cultural and economic contexts are still barriers to the progress of programs that contribute to the decrease of new HIV/AIDS infections and unintended pregnancies among secondary school teens. There are uncertainties in regard to the future of the growing generation, on both sides of claimers, where, some want to protect youth against risky sexual behaviours, while others want to preserve social and cultural norms, which are among the country's identity. The GoR will need to play a key role in putting together concerned groups to get a compromise on this issue to make adolescent reproductive and sexual health a success.

### What is known about this topic

- There is a growing number of unexpected pregnancies among secondary school students, translating into school drop outs, unsafe abortion, early marriages, child abandonment and different health issues associated with early age child bearing including maternal mortality;
- Discussions about providing secondary school with condoms have been initiated to promote safe reproductive and sexual health among secondary school students.

### What this study adds

- Condom distribution in secondary schools evokes different meanings among the debaters, underscoring the complex and messy nature of the condom distribution debate in Rwanda;
- Proponents recognise distributing condoms as a last, needed resort as a solution to protecting and promoting the sexual and reproductive health rights of the youth in secondary schools;
- Opponents argue that adolescents are considered to be underage to be sexually active and providing them with condoms is considered as the lack of societal norms and would mean that parents (and educators) have failed their responsibilities.

## Competing interests

The authors declare no competing interests.
